# Expression of Transposable Elements in the Brain of the *Drosophila melanogaster* Model for Fragile X Syndrome

**DOI:** 10.3390/genes14051060

**Published:** 2023-05-09

**Authors:** Maria Dolores De Donno, Antonietta Puricella, Simona D’Attis, Valeria Specchia, Maria Pia Bozzetti

**Affiliations:** Department of Biological and Environmental Sciences and Technologies, DiSTeBA, University of Salento, Via Monteroni 165, 73100 Lecce, Italy; mariadolores.dedonno@unisalento.it (M.D.D.D.); antonietta.puricella@gmail.com (A.P.); simonadattis83@gmail.com (S.D.); valeria.specchia@unisalento.it (V.S.)

**Keywords:** Fragile X, *Drosophila melanogaster*, transposable elements, piRNA pathway

## Abstract

Fragile X syndrome is a neuro-developmental disease affecting intellectual abilities and social interactions. *Drosophila melanogaster* represents a consolidated model to study neuronal pathways underlying this syndrome, especially because the model recapitulates complex behavioural phenotypes. *Drosophila* Fragile X protein, or FMRP, is required for a normal neuronal structure and for correct synaptic differentiation in both the peripheral and central nervous systems, as well as for synaptic connectivity during development of the neuronal circuits. At the molecular level, FMRP has a crucial role in RNA homeostasis, including a role in transposon RNA regulation in the gonads of *D. m.* Transposons are repetitive sequences regulated at both the transcriptional and post-transcriptional levels to avoid genomic instability. De-regulation of transposons in the brain in response to chromatin relaxation has previously been related to neurodegenerative events in *Drosophila* models. Here, we demonstrate for the first time that FMRP is required for transposon silencing in larval and adult brains of *Drosophila* “loss of function” dFmr1 mutants. This study highlights that flies kept in isolation, defined as asocial conditions, experience activation of transposable elements. In all, these results suggest a role for transposons in the pathogenesis of certain neurological alterations in Fragile X as well as in abnormal social behaviors.

## 1. Introduction

### 1.1. The Drosophila melanogaster Model Exhibits Phenotypical and Molecular Features of Fragile X Syndrome

The *D. m.* genome contains the dFmr1 gene [1], which is homologous with the human *FMR1* gene involved in neurodevelopmental Fragile X syndrome [2]. *Drosophila* “loss of function” dFmr1 mutants have been widely studied due to their exhibiting specific neurological-related phenotypes linked to the human disease. 

Specifically, these mutants exhibit significant olfactory learning impairment, long-term memory defects, and excessive and repetitive grooming [3], as well as abnormal circadian activity and reduced social interactions [4]. *Drosophila* has been definitively established as a model for studying the neurological and behavioural abnormalities caused by Fragile X syndrome [5,6].

A number of the behavioural defects in FraX *Drosophila* mutants have been investigated in order to unveil the pathways underlying the phenotypes. More generally, *dFmr1* mutants show defects in neuronal morphology resembling Fragile X patients’ defects in the brain. dFmr1 is necessary for normal neuronal structure and correct synaptic differentiation in both the peripheral and central nervous systems [7]. Molecular pathways linking Fragile X protein (FMRP) with structural and functional neuronal abnormalities are multiple, and are not completely clarified; however, the role of FMRP as an RNA binding protein is crucial in defining Fragile X phenotypes in humans and in animal models [8]. Multiple RNA targets of FMRP have been identified, both in mammals and *Drosophila*, and FMRP is a component of ribonucleoprotein complexes (RNPs) associated with synaptic polyribosomes [9]. In addition, FMRP is a component of the RNP complexes that transport mRNA along dendrites and axons, and plays a role in the regulation of protein synthesis at the postsynaptic site [10]. The targets of FMRP, namely, RNA and proteins, have an impact on Fragile X phenotypes by altering several cellular processes, such as translation (including through the miRNA pathway) and cytoskeleton remodelling [11,12]. Globally, FMRP has a role in maintaining RNA homeostasis in cells.

### 1.2. dFmr1 Participates in RNA Homeostasis of Transposons in Drosophila melanogaster Gonads

A new role of dFmr1 in RNA homeostasis has been demonstrated for transposons in the gonads of *D. m.* [13,14]. Transposons are repetitive sequences regulated at both the transcriptional and post-transcriptional levels to avoid their spreading into the genomes [15,16]. The regulatory mechanism of transposons has been investigated at both the genetic and molecular levels in *Drosophila* gonads, with a specific class of small non-coding RNAs, Piwi-interacting RNAs (piRNAs), and specific Argonaute family proteins found to be involved [16]. A complex network of proteins has been discovered to act in germline and somatic tissues of the *Drosophila* ovary in piRNA biogenesis and functions [17]. Defects in small RNA-mediated regulation trigger the activation of transposons in germline and somatic tissues in *Drosophila* gonads, thereby generating genomic instability.

*Drosophila* dFmr1 “loss of function” mutants exhibit an abnormally high level of transposon transcripts in both male and female gonads [13,18] along with deregulation of Stellate repetitive sequences [19]. dFmr1 appears to have a very extensive role, acting to regulate transposons at both the transcriptional and post-transcriptional levels. dFmr1 interacts with the Argonaute protein Aubergine, which is involved in post-transcriptional silencing of transposons [13], as well as with another Argonaute protein, Piwi, to maintain heterochromatin and transcriptional silencing in somatic cells of Drosophila ovaries [18]. 

### 1.3. Physiological and Pathological Roles of Transposable Elements in the Nervous System

Emerging evidences suggests an association between the deregulated activation of transposons (TEs) and diseases of the nervous system in *Drosophila* models as well as in human brains [20,21,22].

### 1.4. “piRNA Pathway” in the Nervous System of Drosophila melanogaster and Mouse Models

A simple model of tauopathy in *D. m.* expressing human mutant Tau showed significantly altered levels of transposon transcripts. Heterochromatin decondensation and depletion of piwi/piRNAs have been identified as major mechanistic links between pathogenic tau and loss of transposable element control [22]. 

Dysregulated expression of transposons has been reported in the post-mortem brain tissues of patients with Alzheimer’s disease and progressive supranuclear palsy (another tauopathy) [22,23,24]. Postmortem analysis revealed transposon products in the diseased brains of patients with other neurological disorders causing neurodegeneration: Aicardi-Goutieres syndrome (AGS), Multiple Sclerosis (MS), and Amyotrophic Lateral Sclerosis (ALS). In addition, the *D. m.* model of Huntington’s disease expressing the pathogenic variant of human Huntingtin protein exhibited disregulation of transposons in larval and adult brains [25].

In this paper, we demonstrate for the first time that dFmr1 is required for the silencing of transposons in larval and adult brains of dFmr1 mutants. Our study further displays that, in the brains of flies kept in “asocial” conditions, at least a few transposons are expressed more than in flies grown in normal “social” conditions, thereby demonstrating that “asocial” conditions might influence the regulation of transposons in the brain. 

## 2. Materials and Methods

Fly stocks: We used the genetic wild-type strain *w1118* with a partial deletion of the *white* gene, the *Fmr1*[Delta50M]/*TM6B Tb[+]* (Bloomington Stock Center #6930) strain with hypomorphic allele of *dFmr1,* and the *piwi* [06843]*: P{PZ}piwi^06843^* cn^1^/CyO; *ry^506^* strain (Bloomington Stock Center #12225, Bloomington, IN, USA) with insertion of the element P in the gene *piwi.* Flies were raised on a standard cornmeal agar medium and were maintained at 25 °C.

Total RNA extraction: Total RNA was extracted from adult heads and larval brains (25 males and females flies or larvae for each extraction) using an RNAqueous Micro Kit (Invitrogen, Waltham, MA, USA) and following the manufacturer’s protocol. The RNA concentration and purity were determined photometrically by measuring absorbance at 260 nm with a ratio of A260/A280. To remove all of the DNA in the preparation, samples were incubated with DNase I RNase free at 37 °C for 20 min; after treatment, DNase was inactivated using the DNase Inactivation Reagent from the kit.

cDNA synthesis from total RNA: For first-strand cDNA synthesis, up to 5 micrograms of total RNA was used as a template for oligonucleotide dT(20) primed reverse transcription using the SuperScript III First-Strand Synthesis System (Invitrogen, Waltham, MA, USA) according to the manufacturer’s instructions.

Quantitative real-time PCR: Real-time PCR was performed with the SmartCycler real-time PCR (Cepheid, Sunnyvale, CA, USA) and StepOne Real-Time PCR (Applied Biosystems, Invitrogen, Waltham, MA, USA) systems. Expression of transposable elements was determined by real-time PCR using SsoAdvanced Universal SYBR Green Supermix (Bio-Rad, Hercules, CA, USA) according to the manufacturer’s protocol. For quantification of the transcripts, we used the 2DDct method [26] with rp49 transcripts as control. In the results, the error bars correspond to the standard deviation of three independent experiments. All primers are listed in Table S1.

Statistical analysis: for comparisons between two measurements, a two-tailed Student’s *t*-test was used to show statistical significance.

## 3. Results

### 3.1. Expression of Transposable Elements in Larval Brains and in Heads of dFmr1^−/−^ Mutants

**Figure 1:**
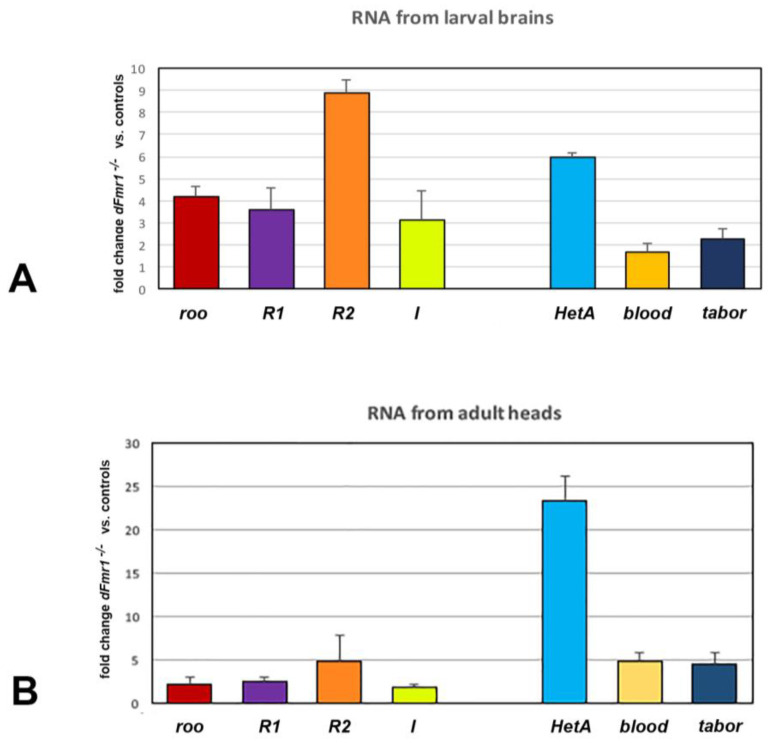
dFMR1D50 mutants have activated transposons in adult heads. qRT-PCR analysis of the indicated transposons in dFMR1^−/−^ homozygous flies versus controls. Data are the mean of three independent experiments, with the error bars calculated as described in the Materials and Methods section. Statistical significance was calculated by applying the *t*-test for both experiments. The *p* value in larval brains, shown in (**A**), is 0.005 for *roo*, 0.034 for *R1*, 0.008 for *R2*, 0.08 for *I*, 0.0002 for *HetA*, 0.058 for *blood*, and 0.003 for *tabor*. The *p* value in adult heads, shown in (**B**), is 0.055 for *roo*, 0.0023 for *R1*, 0.018 for *R2*, 0.0015 for *I*, 0.00002 for *HetA*, 0.00046 for *blood*, and 0.0016 for *tabor*.

*dFmr1* was demonstrated to be a component of the piRNA pathway regulating transposable elements in (at least) fly gonads. Indeed, *dFmr1* mutants exhibited an increase in expression of transposable elements in the gonads, as occurs in RNAi strains in which the *dFmr1* gene is silenced in the testes and ovaries [13]. We demonstrated that *dFmr1* genetically interacts with *aubergine* and *Ago1* at the larval neuromuscular junctions (NMJs). To address the point of *dFmr1* participation in a pathway regulating transposable elements in the nervous system, we used real-time PCR analysis to test the expression of several transposable elements (*roo*, *R1*, *R2*, *I*, *HetA*, *blood*, and *tabor*) in larval brains and heads of *dFmr1D50 ^−^*^/*−*^ and compared the results to *w1118*, used as a control. The graph in Figure 1A shows that the expression of all the transposons reported in the figure increased in larval brains of D50 mutants compared to controls. *R2* and *HetA* exhibit the highest value of fold change in mutant larval brains compared to control brains (8.87 and 5.94 folds), followed by *roo* (4.17 folds), *R1* (3.6 folds), and *I* (3.12 folds) (Figure 1A). In addition, we analysed the expression of the same transposable elements in adult brains of *dFmr1D50 ^−^*^/*−*^ compared to controls in order to test whether the expression of transposable elements is deregulated even in the adult brains, as occurs in the nervous system of third instar larvae. Figure 1B shows that all the TEs tested in the larvae are expressed at a major rate compared to the controls. *Het A* exhibits the highest value compared to controls (23.34 folds), followed by *blood* and *R2* (4.76 and 4.75, respectively). The precise values of the fold changes are shown in Table S1 for larval brains and adult heads. We analysed other transposons as well (*412*, *springer*, and *thare*), which do not increase in comparison to the controls.

In order to support the hypothesis that the piRNA pathway is involved in the regulation of transposons in the nervous system, we tested the expression of several TEs in larval and adult brains of piwi mutants. We observed that *Het A* expression increased in both adult and larval brains (Figure S1), with major expression in the adults.

The experiments reported above demonstrate that correct functioning of *dFmr1* in larval brains and adult heads is required for the silencing of at least some transposable elements as well as that functional piwi is required for *Het A* regulation, suggesting that certain neurological phenotypes of *dFmr1* mutants might be related to TE-mediated genome instability in the nervous tissues.

### 3.2. Expression of TEs in Wild-Type Flies Grown in Different “Social” Conditions

**Figure 2:**
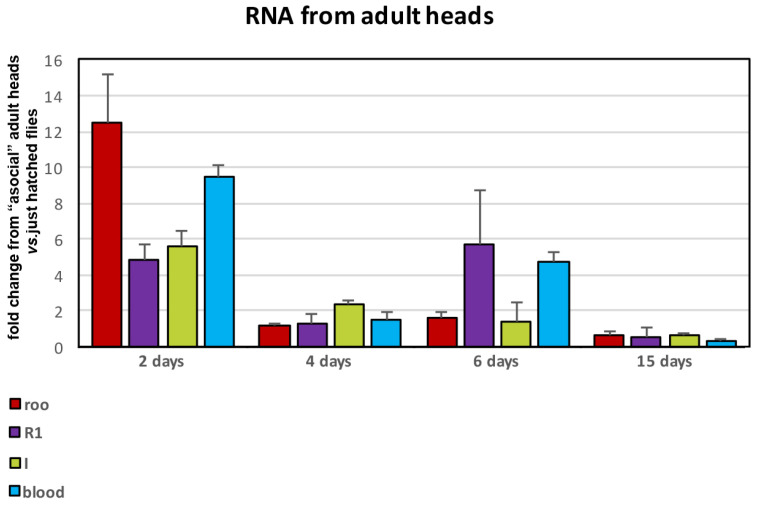
Wild-type flies grown in isolation possess activated transposons in adult heads. qRT-PCR analysis of expression of the indicated transposons in wild-type/Oregon R flies grown in isolation (asocial condition) compared to flies grown in social condition. Data are the mean of three independent experiments. Error bars were calculated as described in the Materials and Methods section. Statistical significance was calculated by applying *t*-tests for all the experiments. The *p* value for the brains of flies kept isolated for 2 days was 0.0233 for *roo*, 0.0237 for *R1*, 0.011 for *I*, and 0.0044 for *blood*. The *p* value for the brains of flies kept isolated for 4 days was 0.0089 for *roo*, 0.095 for *R1*, 0.0346 for *I*, and 0.0042 for *blood*. The *p* value for the brains of flies kept isolated for 6 days was 0.00235 for *roo*, 0.0315 for *R1*, 0.422 for *I*, and 0.037 for *blood*. The *p* value for the brains of flies kept isolated for 15 days was 0.028 for *roo*, 0.070 for *R1*, 0.026 for *I*, and 0.017 for *blood*.

In addition to classical FraX phenotypes, Fragile-X syndrome patients often exhibit autism spectrum disorders (ASDs) such as impaired social interactions, repetitive stereotyped/fixed behaviours, and language and sensory anomalies. Therefore, we focused our attention on social interactions in wild-type flies. We evaluated the effect of different “social” conditions on the expression of transposable elements in the flies’ heads. Recently hatched wild-type flies were transferred into tubes and grown separate from their “community”, being completely alone for 2, 4, 6, and 15 days. After that, heads from flies subjected to a specific period of isolation were collected and their RNA was analysed by real time PCR for the expression of selected TEs (*roo*, *R1*, *I*, and *blood*), then compared with flies grown in the “community”, that is, in normal conditions (Figure 2). After two days in the “asocial” condition, the expression of almost all the assayed transposable elements increased with respect to flies grown in the normal “social” condition. On the fourth day, levels of the TEs expression were similar between the two conditions. On the sixth day, certain transposable elements (*R1* and *blood*) increased again, finally reaching lower levels with respect to the flies grown in the community on the fifteenth day. The precise values of the fold changes are shown in Table S2 for adult heads. These results suggest that flies grown alone in “asocial” conditions have transposable elements in their brains after as little as 2 days, and that this activation might have effects on the correct development of the nervous system related to behavioural phenotypes, including those related to social interaction.

## 4. Discussion

Molecular mechanisms underlying Fragile X syndrome, a rare neurological disease affecting intellectual abilities frequently associated with autism spectrum disorders and epilepsy, have been described previously [27], even though not all of the pathways potentially involved in FraX syndrome have been explored. *D. m.* represents a consolidated and attractive system for studying Fragile X disease in detail. It has been widely used to investigate *FMR1* functions at the molecular, genetic, and cellular levels, as *Drosophila Fmr1* mutants exhibit several FXS phenotypes [6]. In the *Drosophila* model, FMRP is required for experience-dependent changes in synaptic connectivity during the development of the neuronal circuits for sensory input as well as in regulating neuron-to-glia communication in neuronal circuits [28]. A role of *Drosophila* FMRP was demonstrated in the regulation of TEs in the gonads [13]. In this paper, we have demonstrated for the first time that at least some TEs are activated in larval as well as adult brains of *dFmr1D50^−^*^/*−*^ mutants (previously demonstrated to be a “loss of function” mutant [13]). We demonstrated that both “germline” and “somatic” transposons (*roo*, *R1*, *R2*, *I*, *HetA*, *blood*, and *tabor*) are expressed more than in controls in both larval and adult brains. *R2* and *HetA* are the most expressed in larval brains, while *Het A* is the most expressed in adult brains and piwi mutants. These results suggest that transposable elements are deregulated in the larval and adult nervous system of *dFmr1* mutants, as occurs in the *dFmr1*^−/−^gonads [13]. The reported experiments demonstrate that correct functioning of *dFmr1* in larval brains and adult heads is required for the silencing of at least some, transposable elements, suggesting that a number of the neurological phenotypes of *dFmr1* mutants might be related to TE-mediated genome instability in the nervous tissues. These results allow us to propose that a pathway regulating the transposable elements is active in the nervous system to silence TEs, which probably means that the piRNA pathway (or a pathway with similar features that uses small RNA to silence the transposons) has a role in these tissues and participates in the neurological phenotypes observed in *dFmr1*^−/−^ mutants. A physiological role of TEs in neuronal genetic plasticity and during the development of the nervous system of both vertebrates and invertebrates has previously been reported [29,30]. In any event, the presence of the piRNA pathway in the brains of different organisms remains controversial [31]. However, pan-neuronal silencing of piwi-elevated expression of TEs in the brains of tau-transgenic flies corroborates the hypothesis that the piRNA pathway is the main contributor to TE expression [22]. Furthermore, transposon sequences are mainly located in the heterochromatin, which ensures their transcriptional silencing. These repetitive sequences are repressed by H3K9me2/3, the epigenetic marker for Heterochromatin Protein 1 (HP1) [32,33]. Heterochromatin relaxation is emerging as a crucial mechanism underlying the activation of transposons in the brains of animal models during neurological disorders. In brains of tau-transgenic *Drosophila* as well as in models of Alzheimer’s disease, heterochromatin decondensation causes activation of transposons, including *copia*, *gypsy*, and *HetA* [21]. Additionally, in larval and adult *Drosophila* brains expressing human mutant Huntingtin, heterochromatin loss drives the deregulation of transposons, with *HetA* being the most induced class at the larval stage [25]. In neurons, the stability of HP1 and heterochromatin structure depends on the correct organization of the nuclear lamin. It has been demonstrated that the loss of nuclear lamin organization is responsible for chromatin relaxation, and consequently for transposon activation in neurons [24,34]. This mechanism of transposon activation has been related to neurodegenerative events, and could be involved in other neuropathological diseases. Our results support the hypothesis that deregulation of TEs in the central brain might contribute to neurological phenotypes in a *dFmr1^−^*^/*−*^ mutant fly model of Fragile X Syndrome. This research opens up the possibility of investigating the role of FMRP in the control of transposons in the brain of mouse models of Fragile X Syndrome

Fruit flies are considered to be social animals; They interact with each other in many activities, such as sharing information about food availability, searching for partners, and synchronizing daily activities. Social interactions might affect their behaviour, though this is also dependent on age, sex, and genetics [35,36,37]. In certain neurological disorders, including Fragile X syndrome, the most common form of ID frequently associated with autism, patients exhibit altered social behaviours. Indeed, *dFmr1* mutants interact with each other less often than wild-type flies, and fail to initiate social interactions, thereby increasing the distance among flies [4]; this suggests that these mutants have important social defects.

To summarise, in this paper we investigated the relation between the “asocial” vs. “social” condition in wild-type flies and the regulation of transposable elements in fly and larval brains. Our results demonstrate that animals grown in “asocial” conditions exhibit activation of certain transposable elements (*roo*, *R1*, *I*, and *blood*) as evaluated at different days with respect to animals grown in normal “social” conditions for exactly the same period. Most activation of TEs occurs after 2 days in the “asocial” condition (Figure 2), while the differences between the two conditions are lessened over time. We suggest that the “asocial” condition might act as a stress condition that deregulates the transposable elements in wild-type flies, and that this effect is already present after two days of isolation. The activation of transposons has previously been reported in *Drosophila* and other organisms in response to different types of stress, including repeated restraint stress in mice [38]. Further analysis of this phenotype in mutants such as *dFMR1^−^*^/*−*^ flies can contribute to understanding the role, if any, of transposable elements in correct development of the neurological tissues as well as the effects on the social behaviour in animal models of autism spectrum disorders.

Both of the results reported in this contribution represent neurological phenotypes that can be used to test genes and pathways related to *dFmr1*, and could be useful in evaluating the effect of new therapeutic molecules or treatments for neurological defects.

## Data Availability

Not applicable.

## Supplementary Materials

The following supporting information can be downloaded at: https://www.mdpi.com/article/10.3390/genes14051060/s1, Figure S1: piwi mutants activate HetA transposon in adult heads and larval brains. qRT-PCR analysis of the indicated transposons in piwi mutants homozygous flies versus controls. Data are mean from three independent experiments; error bars have been calculated as described in the Materials and Methods. The results calculated by applying the T-Test for the experiments are statical significative; Table S1: fold changes and standard deviations reported in graphs of Figure 1A (larval brains) and Figure 1B (adult brains); Table S2: fold changes and standard deviations reported in graphs of Figure 2.

## References

[1] Wan L., Dockendorff T.C., Jongens T.A., Dreyfuss G. (2000). Characterization of DFMR1, a *Drosophila melanogaster* Homolog of the Fragile X Mental Retardation Protein. Mol. Cell. Biol..

[2] Bardoni B., Mandel J.L., Fisch G.S. (2000). FMR1 Gene and Fragile X Syndrome. Am. J. Med. Genet..

[3] Bolduc F.V., Bell K., Cox H., Broadie K.S., Tully T. (2008). Excess Protein Synthesis in Drosophila Fragile X Mutants Impairs Long-Term Memory. Nat. Neurosci..

[4] Bolduc F.V., Valente D., Nguyen A.T., Mitra P.P., Tully T. (2010). An Assay for Social Interaction in Drosophila Fragile X Mutants. Fly.

[5] Bardoni B., Capovilla M., Lalli E. (2017). Modeling Fragile X Syndrome in Neurogenesis: An Unexpected Phenotype and a Novel Tool for Future Therapies. Neurogenesis.

[6] Specchia V., Puricella A., D’Attis S., Massari S., Giangrande A., Bozzetti M.P. (2019). Drosophila melanogaster as a Model to Study the Multiple Phenotypes, Related to Genome Stability of the Fragile-X Syndrome. Front. Genet..

[7] Zhang Y.Q., Bailey A.M., Matthies H.J., Renden R.B., Smith M.A., Speese S.D., Rubin G.M., Broadie K. (2001). Drosophila Fragile X-Related Gene Regulates the MAP1B Homolog Futsch to Control Synaptic Structure and Function. Cell.

[8] O’Donnell W.T., Warren S.T. (2002). A Decade of Molecular Studies of Fragile X Syndrome. Annu. Rev. Neurosci..

[9] Brown V., Jin P., Ceman S., Darnell J.C., O’Donnell W.T., Tenenbaum S.A., Jin X., Feng Y., Wilkinson K.D., Keene J.D. (2001). Microarray Identification of FMRP-Associated Brain MRNAs and Altered MRNA Translational Profiles in Fragile X Syndrome. Cell.

[10] Broadie K.S., Richmond J.E. (2002). Establishing and Sculpting the Synapse in Drosophila and C. Elegans. Curr. Opin. Neurobiol..

[11] Schenck A., Bardoni B., Langmann C., Harden N., Mandel J.L., Giangrande A. (2003). CYFIP/Sra-1 Controls Neuronal Connectivity in Drosophila and Links the Rac1 GTPase Pathway to the Fragile X Protein. Neuron.

[12] Caudy A.A., Myers M., Hannon G.J., Hammond S.M. (2002). Fragile X-Related Protein and VIG Associate with the RNA Interference Machinery. Genes Dev..

[13] Bozzetti M.P., Specchia V., Cattenoz P.B., Laneve P., Geusa A., Sahin H.B., Di Tommaso S., Friscini A., Massari S., Diebold C. (2015). The Drosophila Fragile X Mental Retardation Protein Participates in the PiRNA Pathway. J. Cell. Sci..

[14] Specchia V., D’Attis S., Puricella A., Bozzetti M.P. (2017). DFmr1 Plays Roles in Small RNA Pathways of Drosophila Melanogaster. Int. J. Mol. Sci..

[15] Pimpinelli S., Berloco M., Fanti L., Dimitri P., Bonaccorsi S., Marchetti E., Caizzi R., Caggese C., Gatti M. (1995). Transposable Elements Are Stable Structural Components of Drosophila Melanogaster Heterochromatin. Proc. Natl. Acad. Sci. USA.

[16] Malone C.D., Brennecke J., Dus M., Stark A., McCombie W.R., Sachidanandam R., Hannon G.J. (2009). Specialized PiRNA Pathways Act in Germline and Somatic Tissues of the Drosophila Ovary. Cell.

[17] Czech B., Munafò M., Ciabrelli F., Eastwood E.L., Fabry M.H., Kneuss E., Hannon G.J. (2018). PiRNA-Guided Genome Defense: From Biogenesis to Silencing. Annu. Rev. Genet..

[18] Jiang F., Lu F., Li P., Liu W., Zhao L., Wang Q., Cao X., Zhang L., Zhang Y.Q. (2016). Drosophila Homolog of FMRP Maintains Genome Integrity by Interacting with Piwi. J. Genet. Genom..

[19] Bozzetti M.P., Fanti L., Di Tommaso S., Piacentini L., Berloco M., Tritto P., Specchia V. (2012). The “Special” *Crystal-Stellate* System in *Drosophila Melanogaster* Reveals Mechanisms Underlying PiRNA Pathway-Mediated Canalization. Genet. Res. Int..

[20] Krug L., Chatterjee N., Borges-Monroy R., Hearn S., Liao W.-W., Morrill K., Prazak L., Rozhkov N., Theodorou D., Hammell M. (2017). Retrotransposon Activation Contributes to Neurodegeneration in a Drosophila TDP-43 Model of ALS. PLoS Genet..

[21] Guo C., Jeong H.-H., Hsieh Y.-C., Klein H.-U., Bennett D.A., De Jager P.L., Liu Z., Shulman J.M. (2018). Tau Activates Transposable Elements in Alzheimer’s Disease. Cell. Rep..

[22] Sun W., Samimi H., Gamez M., Zare H., Frost B. (2018). Pathogenic Tau-Induced PiRNA Depletion Promotes Neuronal Death through Transposable Element Dysregulation in Neurodegenerative Tauopathies. Nat. Neurosci..

[23] Ravel-Godreuil C., Znaidi R., Bonnifet T., Joshi R.L., Fuchs J. (2021). Transposable Elements as New Players in Neurodegenerative Diseases. FEBS Lett..

[24] Ramirez P., Zuniga G., Sun W., Beckmann A., Ochoa E., DeVos S.L., Hyman B., Chiu G., Roy E.R., Cao W. (2022). Pathogenic Tau Accelerates Aging-Associated Activation of Transposable Elements in the Mouse Central Nervous System. Prog. Neurobiol..

[25] Casale A.M., Liguori F., Ansaloni F., Cappucci U., Finaurini S., Spirito G., Persichetti F., Sanges R., Gustincich S., Piacentini L. (2021). Transposable Element Activation Promotes Neurodegeneration in a Drosophila Model of Huntington’s Disease. iScience.

[26] Livak K.J., Schmittgen T.D. (2001). Analysis of Relative Gene Expression Data Using Real-Time Quantitative PCR and the 2(-Delta Delta C(T)) Method. Methods.

[27] Bagni C., Zukin R.S. (2019). A Synaptic Perspective of Fragile X Syndrome and Autism Spectrum Disorders. Neuron.

[28] Song C., Broadie K. (2023). Fragile X Mental Retardation Protein Coordinates Neuron-to-Glia Communication for Clearance of Developmentally Transient Brain Neurons. Proc. Natl. Acad. Sci. USA.

[29] Coufal N.G., Garcia-Perez J.L., Peng G.E., Yeo G.W., Mu Y., Lovci M.T., Morell M., O’Shea K.S., Moran J.V., Gage F.H. (2009). L1 Retrotransposition in Human Neural Progenitor Cells. Nature.

[30] Baillie J.K., Barnett M.W., Upton K.R., Gerhardt D.J., Richmond T.A., De Sapio F., Brennan P.M., Rizzu P., Smith S., Fell M. (2011). Somatic Retrotransposition Alters the Genetic Landscape of the Human Brain. Nature.

[31] Treiber C.D., Waddell S. (2017). Resolving the Prevalence of Somatic Transposition in Drosophila. eLife.

[32] Fanti L., Pimpinelli S. (2008). HP1: A Functionally Multifaceted Protein. Curr. Opin. Genet. Dev..

[33] Filion G.J., van Bemmel J.G., Braunschweig U., Talhout W., Kind J., Ward L.D., Brugman W., de Castro I.J., Kerkhoven R.M., Bussemaker H.J. (2010). Systematic Protein Location Mapping Reveals Five Principal Chromatin Types in Drosophila Cells. Cell.

[34] Napoletano F., Bravo G.F., Voto I.A.P., Santin A., Celora L., Campaner E., Dezi C., Bertossi A., Valentino E., Santorsola M. (2021). The Prolyl-Isomerase PIN1 Is Essential for Nuclear Lamin-B Structure and Function and Protects Heterochromatin under Mechanical Stress. Cell. Rep..

[35] Huang R., Song T., Su H., Lai Z., Qin W., Tian Y., Dong X., Wang L. (2020). High-Fat Diet Enhances Starvation-Induced Hyperactivity via Sensitizing Hunger-Sensing Neurons in Drosophila. eLife.

[36] Chen J., Jin S., Chen D., Cao J., Ji X., Peng Q., Pan Y. (2021). Fruitless Tunes Functional Flexibility of Courtship Circuitry during Development. eLife.

[37] Chen M., Sokolowski M.B. (2022). How Social Experience and Environment Impacts Behavioural Plasticity in Drosophila. Fly.

[38] Cappucci U., Torromino G., Casale A.M., Camon J., Capitano F., Berloco M., Mele A., Pimpinelli S., Rinaldi A., Piacentini L. (2018). Stress-Induced Strain and Brain Region-Specific Activation of LINE-1 Transposons in Adult Mice. Stress.

