# Supplementary material for: Expression of Transposable Elements in the Brain of the Drosophila melanogaster Model for Fragile X Syndrome
Source: Genes (Basel). 2023 May 9;14(5):1060. doi: 10.3390/genes14051060 (PMC10218032; doi:10.3390/genes14051060)
Supplement: Supplementary file 1 [file genes-14-01060-s001.zip › genes-2343644-supplementary.pdf]

Table S1. fold changes and standard deviations reported in graphs of Figure 1A (larval brains) and Figure 1B (adult brains)

RNA from larval brains

	roo	*R1*	*R2*	*I*	*HetA*	*blood*	*tabor*
**fold change**	4,17	3,6	8,87	3,12	5,94	1,70	2,26
**st. dev.**	0,46	1,01	0,6	1,36	0,22	0,37	0,46

RNA from adult brains

	roo	*R1*	*R2*	*I*	*HetA*	*blood*	*tabor*
**fold change**	2,04	2,43	4,75	1,73	23,34	4,76	4,4
**st. dev.**	0,87	0,43	2,93	0,27	2,73	1,04	1,29

Table S2. fold changes and standard deviations reported in graphs of Figure 2

RNA from adult heads

	roo	**st.dev. *roo***	*R1*	**st.dev. *R1***	*I*	**st.dev. *I***	*blood*	**st.dev. *blood***
**fold change 2 days**	12,5	2,63	4,82	0,84	5,635	0,71	9,435	0,79
**fold change 4 days**	1,21	0,02	1,33	0,46	2,36	0,36	1,51	0,25
**fold change 6 days**	1,64	0,33	5,73	2,97	1,40	1,05	4,715	0,16
**fold change 15 days**	0,69	0,18	0,55	0,49	0,66	0,19	0,28	0,13

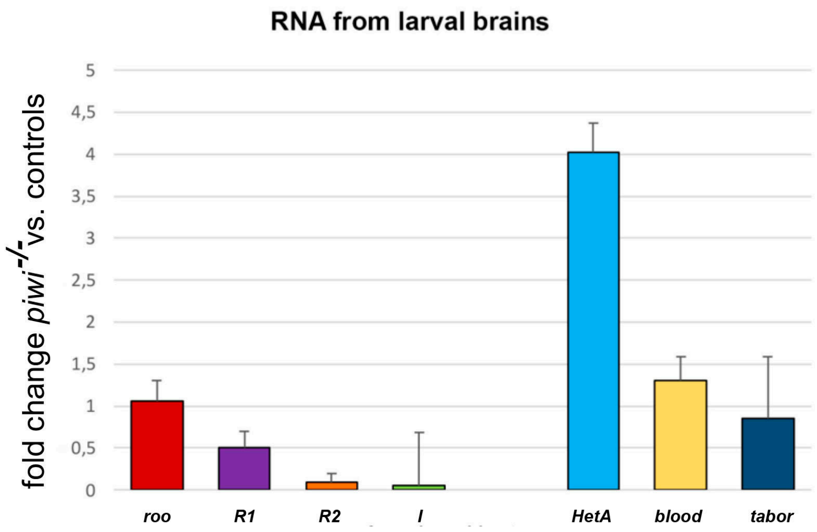

RNA from larval brains

	roo	*R1*	*R2*	*I*	*HetA*	*blood*	*tabor*
fold change	1,06	0,51	0,99	0,06	4,02	1,3	0,85
st. dev.	0,25	0,19	0,11	0,63	0,35	0,29	0,74

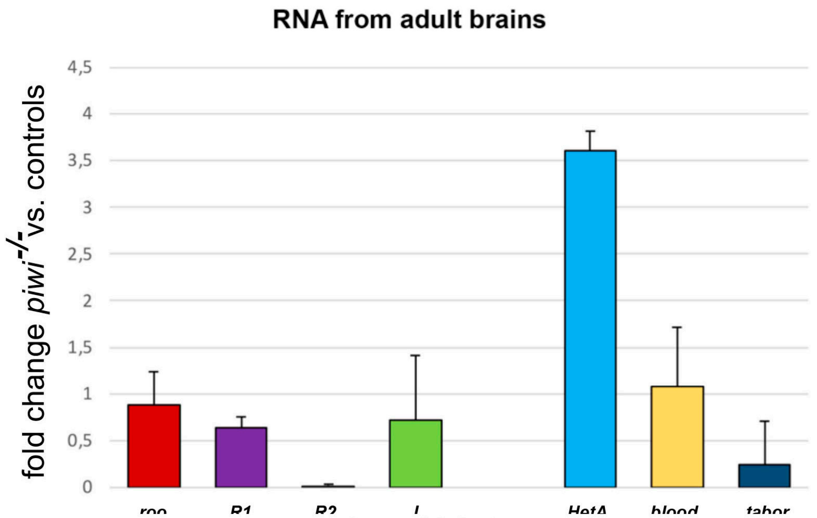

RNA from adult brains

	roo	*R1*	*R2*	*I*	*HetA*	*blood*	*tabor*
fold change	0,88	0,64	0,01	0,72	3,6	1,08	0,24
st. dev.	0,36	0,11	0,023	0,69	0,21	0,64	0,47

Legend to Figure S1

Figure S1. *piwi* mutants activate HetA transposon in adult heads and larval brains. qRT-PCR analysis of the indicated transposons in *piwi* mutants homozygous flies versus controls. Data are mean from three independent experiments; error bars have been calculated as described in the Materials and Methods. The results calculated by applying the T-Test for the experiments are statical significative.
